# Modelling the utilization rates of pedestrian crosswalks

**DOI:** 10.1016/j.heliyon.2023.e19310

**Published:** 2023-08-19

**Authors:** Walid Abdullah Al Bargi, Basil David Daniel, Nasradeen A. Khalifa, Munzilah Md Rohani, Qinaat Hussain, Rafidah Binti Hamdan

**Affiliations:** a Advanced Pavement Material (PAVE-MAT) Focus Group, Faculty of Civil Engineering and Built Environment, Universiti Tun Hussein Onn Malaysia, 86400, Parit Raja, Batu Pahat, Johor, Malaysia; b Smart Driving Research Centre, Faculty of Civil Engineering and Built Environment, Universiti Tun Hussein Onn Malaysia, 86400, Parit Raja, Johor, Batu Pahat, Malaysia; c Qatar Transportation and Traffic Safety Center, College of Engineering, Qatar University, PO Box 2713, Doha, Qatar

**Keywords:** Pedestrians, Zebra crossing, Utilization rate, Logistic regression, Malaysia

## Abstract

A zebra crossing is a form of pedestrian crossing provision point on roads that have significant volumes of traffic. It is one of the safety measures employed to reduce avoidable pedestrian-motorist conflicts and accidents on such roads. In the past several studies have been conducted on the behaviours of road users (pedestrians and motorists) especially on non-signal intersections. Although, various recommendations and solutions have been proffered to the many road-crossing challenges. But there has been little to no change in pedestrians’ perceptions and preferences of zebra crossing. Contemporary researches have tried to rectify this by applying several models to rate the use of pedestrian zebra crossing. This study uses a Regression Model Techniques to analyse factors influencing utilization rate of pedestrian zebra crossing. In this study, 450 samples were collected from 12 locations, covering school, public building, residential and business areas to examine the utilization rate of the zebra crossing by pedestrians. To examine the significance level on the crossing utilization rates by pedestrian at 95% confidence interval, a pedestrian utilization rate (PUR) was acting as the dependent variable and the remaining variables served as the independent variables. The Multiple Linear Regression (MLR) model was also used to determine the utilization rate needed to develop the zebra crossing utilization model. From the findings, the calibrated R2 value was discovered to be at 0.937 and the descriptive statistics of MLR test, t and p-values, were also found within an acceptable range. The result also showed that, out of all the variables which were used, only three have a significant effect on the utilization rates of pedestrian zebra crossing while the remaining variables have an insignificant effect. The study concluded that among the different variables, Guardrail, number of lanes and Width of zebra crossing were the most influential variables. It is believed that the conclusions drawn from this research are expected to be useful to improve the state of pedestrian facilities in Malaysia.

## 1 Introduction

The increasing population in urban areas has resulted in a surge in traffic congestion, which has prompted concerns about the safety of road users, especially pedestrians. According to a report by the United Nations, the global urban population is expected to increase to 68% by 2050, with most of the growth occurring in Africa and Asia [1]. This increase in urbanization has resulted in overcrowding and increased demand for public transportation and personal vehicles. In most densely populated cities, it has been discovered that the number of pedestrians at every point in time is more than that of other road users. It is why such roads are susceptible to traffic congestions if pedestrian traffic is not properly managed [2]. Taking Malaysia as a study case, many cities have recorded bad crosswalk road-utilization. This is due to the likely tendency of pedestrians to violate traffic rules and regulations when crossing, although a lack of necessary crossing facilities can also be said to be responsible [3]. This high record of bad crosswalk road-utilization has directly led to a large proportion of pedestrian casualties in road accidents. This is why there is the need for more crossing facilities such as zebra crossings, which must carry high friction surfacing on several approaches [4]. It must be noted that such facilities must be installed only after proper economic evaluation has been carried out to determine the cost effectiveness of such projects. Such facilities previously installed have been found many times to be very poorly utilized and cost ineffective. Such economic waste will be guarded against if, as stated, proper economic procedures are followed.

Pedestrian safety is a crucial aspect of urban planning and transportation infrastructure development. Providing crossing facilities for pedestrians is a common strategy to enhance pedestrian safety [5]. However, special care must be taken to ensure that such facilities are designed and implemented in a way that minimizes the risk of pedestrian accidents. Several measures (barriers, escalators, and ramps) have to be introduced to discourage and prevent illegal pedestrian crossings as being done in many other countries. One of the reasons for the illegal crossing tendencies of most pedestrians is the inconvenience attached to the crossing points, mostly a result of error in design. In Malaysia, another of such reasons is the absence of specific warrant or necessity for using zebra crossing excluding the common measures provided to deter less utilization. The common measures that serve as the rules and regulations was published by Jabatan Kerja Raya (JKR) on pedestrian facilities. It is called Nota Teknik Jalan 18/97 received from AUSTROADS (Australia) Guide to Traffic Engineering Practice, Part 13-Pedestrian (1995), which was adopted temporarily and subjected to necessary changes [6]. Even with the provision of this regulations (the functional classification of road, the character of the locality, the number and characteristic of pedestrian, the road condition and vehicular traffic condition), as result of the low usage of pedestrian zebra crossing in Malaysia, there is yet the need for a thorough understanding on the utilization rates. Therefore, this study intends to fill the gap by focusing on utilization rate and pedestrians’ behaviours.

## 2 Literature review

This section emphasizes the analysis of the main body of the extant literature namely the pedestrian utilization of crossing facility.

### 2.1 Factors affect pedestrian crossing choice

Pedestrian crossing choice is influenced by a variety of factors, including infrastructure, environmental factors, social factors, and individual characteristics. When it comes to signalized crosswalks, pedestrian crossing choice is primarily affected by the signal timing, pedestrian volume, and vehicle volume. Signal timing is a crucial factor that determines when and how pedestrians cross the street. According to Ref. [7], pedestrian crossing behavior at signalized intersections is strongly influenced by the pedestrian signal timing. The study found that longer pedestrian signal timing increased the likelihood of pedestrians crossing during the walk phase, while shorter pedestrian signal timing led to more pedestrians crossing during the flashing don't walk phase. Pedestrian volume is another important factor that influences crossing behavior at signalized crosswalks. As the volume of pedestrians increases, the risk of conflict between pedestrians and vehicles also increases [8]. found that pedestrian crossing behavior at signalized intersections was significantly influenced by pedestrian volume. The study found that pedestrians tended to cross during the walk phase when the pedestrian volume was high. Vehicle volume is also a critical factor that affects pedestrian crossing behavior at signalized crosswalks. According to Ref. [2], pedestrian crossing behavior is influenced by the number of vehicles waiting at the intersection. The study found that when there were more vehicles waiting at the intersection, pedestrians tended to be more cautious when crossing.

In addition to these primary factors, several other factors can also influence pedestrian crossing behavior at signalized crosswalks, including pedestrian age, gender, and destination, as well as weather conditions and time of day. Understanding these factors can help in designing more effective and safer signalized crosswalks [9]. [10] reported that signalized intersections significantly contributed to pedestrians engaging in illegal crosswalk behavior. The study suggested that proper signalization at intersections would optimize road utilization. However, it should be noted that the research only examined narrow roads where vehicles maintained a low speed limit, which limited the generalizability of the findings. Although this may not be applicable to many roads. Moreover, one of the reason of pedestrians violation of traffic regulations as stated by Ref. [11] is the length of waiting time (45∼60s) of signal light when crossing road. They also suggested more factors such as road width, traffic volume, and pedestrian psychology etc [12].

Some other literature stated, otherwise, that the choice of pedestrians while crossing road could be a result of other several factors such as condition, location, perceptions and design of the facilities at the intersection [[13], [14], [15], [16]]. According to some studies, one of the most influential factors for pedestrian's decision to cross at a stated area is the difference between the location of the facility and pedestrian destination [17,18]. Their research found that pedestrians preferred routes which are closer or faster to their destination. Convenience of crossing facility is another emphasis made by some other researchers [19]. They believed that these serves as motivations for pedestrians to utilize the crossing facilities provided. Thus, if providing these facilities, diligent consideration of the preference of the pedestrian is off important else might create low utilization of the crossing aid. Wrong locations of the crossing facilities also reduce pedestrian's choice of using this facility. Some other researchers stated that, pedestrians are likely to utilize crossing facilities located on major roads than those on minor roads because of density and high volume of traffic [2]. Other factors they identified which influence pedestrian crossing decision process includes the availability of barriers and vegetation, coloured paving and shelter, the timing of signals and configuration of the centers along the road are said to be influencing pedestrian's behavior and compliance rate when utilizing these facilities [20]. Lastly, Regional distribution, economics levels, city scale and culture characteristics also contributed to the behaviours of pedestrians towards utilizing these facilities [6].

### 2.2 Safety measures as a concern for the crossing attitude or behavior of pedestrians

Numerous incidents of pedestrian fatalities and injuries in road accidents have raised alarm about pedestrian safety, prompting the need for enhanced road safety measures to improve the safety of pedestrians [21]. The uninformed actions of many pedestrians make them more vulnerable than other road users, and again play a major role in their fatality and injuries in accidents [22,23]. Actions of the Pedestrian such as inappropriate crossing before the traffic signals, illegal crossing outside designated crosswalk signs are all risky for the pedestrians, thus, the need for adequate measures [24]. These high inappropriate crosswalk behaviours can also vary with gender of the pedestrians. Greater risk taking tendencies of the male gender such as (non-compliance with traffic laws, walking in the dark, tolerance for crossing within narrow gaps, walking under the influence of drug or alcohol) make them more vulnerable to this high risk than their female counterpart [25]. Also, horridness and the general desire of pedestrians to keep moving was one of the foremost factors behind the lack of compliance.

Several research studies have been carried out to address the forgoing concerns of pedestrian safety that are related to their crossing attitude and behaviours [[26], [27], [28]]. One of such studies can be credited to Ref. [29] that focused on development of a model to estimate pedestrian waiting time and serve as a safe guard while crossing section of a road. In his research, many factors determined pedestrian behaviours at crossing sections. This factors include crossing frequency, population of the pedestrians cross in time, gender, age, pedestrian destination, private vehicle accessibility and experience of the pedestrians in previous traffic accidents. Another model developed by Ref. [30] on pedestrian crossing attitude or behavior was to assess the possibility of accident along pedestrian journey using nested logit model and linear regression model. Their findings showed that the probability of accident on a particular crossing area will depend on the crossing options adopted by pedestrians when crossing. Furthermore, another model was carried out by Refs. [[31], [32], [33]] to investigate different numbers of faults in vehicle crashes of pedestrians. Their result revealed that pedestrians’ faults was about 59% of the crashes, drivers at 32%, and both were at fault at 9%. Other inappropriate behaviours associated with pedestrian crossing and contributing to factors of accidents on road include pedestrians not obeying traffic rules and regulations, jaywalking and walking under the influence of drug or alcohol [34].

Some factors, however, including utilization rates of crossings facility and type of vehicle, among others, are under investigation and need further research. For example, search results of pedestrian utilization of crossing facilities in the ScienceDirect/or Scopus database, with “the utilization rates of pedestrian zebra crossing” in the article title, abstract and/or keywords of indexed articles, showed there has been no detailed investigation on usage of zebra crossing, only a little published data. Also, some of these identified factors have different characteristics, operational and traffic conditions, and work environment that are unique to their selected or study areas [6,14,35,36]. Thus, there is a need to consider the different condition and road environment in Malaysia.

To address the identified gaps, this research focuses on the development of a pedestrian model based on traffic flow density along urban streets in Malaysia, which also covers pedestrian perceptions and behaviours in order to provide a safer and more acceptable ambiance for road users. This will, indeed, be useful for engineers and planners when working on crossing infrastructures in the near future.

## 3 Materials and method

**Fig. 1:**
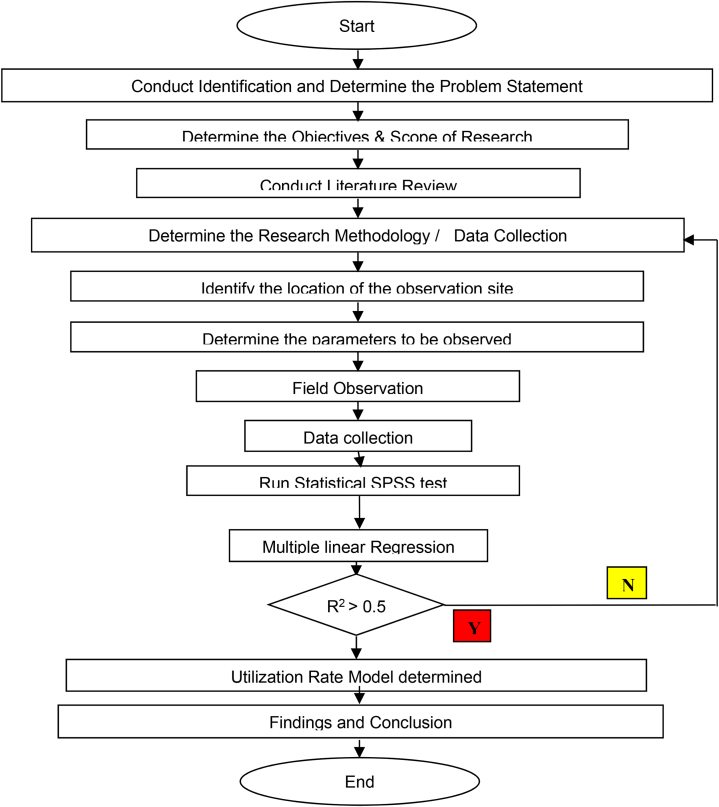
Flowchart of a research process.

In order to achieve this research stated objective, the use of both qualitative and quantitative approaches was employed. For data collection, after several studies and analysis of the literature review, Multiple Linear Regression (MLR) methods was adopted for analyses. Data collections, model development and statistical tests are all showed in Fig. 1.

## 4 Instruments for data collection

Adequate information for this research was gathered through the selection of different sites classified in to four categories to have a full evaluation of pedestrians’ behaviours towards zebra crossing. These categories include the following environments (Amenities, business, residential, and schools) and 12 pedestrian Zebra crossings were observed out of them. The main reason for these 12 selected locations was to measure the level of utilization of available facilities. Videotaping was employed to observe the zebra crossing utilization by pedestrians. Several instruments such as video camera, measuring tape and stopwatch was used for data collection.

## 5 Data collection and model development

In other to analyse factors influencing the usage of pedestrian zebra crossing, data of pedestrians' behaviours were employed. In this research, the pedestrians who used the zebra crossing option while crossing the road section represent the percentage of utilization rate. Video recording called “video data method” was used to retrieve data on pedestrians’ behaviours. This was possible because of the many advantages it has over the manual method. The likes of low cost economic advantage of running video survey on a permanent and continuous record of events makes it more preferable. Also this method was used because, it was very easy and faster to observe within an hour and obtain directly the total number of pedestrians who desired to utilize the zebra crossing compared to those who did eventually. Number of the pedestrians was one of the essential data for this research. The reason was that the volume of pedestrian needed for the study compared to what was available at each site location was lower, hence the need for proper and adequate figure.

Lastly, the use of the videotape was targeted to gather information on pedestrians who crossed the road. The following categories of pedestrians were not counted in the samples of this research such as those waiting for the bus and not having intention of crossing, the once walking on the roadside, and those who were done crossing from a distance higher than 50 m.

### 5.1 Statistical Package for Social Science software (SPSS)

Computation of the descriptive statistics including standard error, t-value, and p-value, under Multiple Linear Regression, Statistical Package for Social Science software (SPSS) version 22 was employed to carry out data analysis. The general functions and steps in Multiple Linear Regression were used for analysis of the data.

### 5.2 Multiple linear regression model (MLR model)

A multiple linear regression (MLT) analysis was carried out to analyse the contributing factors to the utilization rates of pedestrian zebra crossing. Results showed that 3 out of 5 variables that have been analysed were significant in its association with the pedestrian Zebra crossing utilization. The general model framework is given by Equation (1) below:

(1) PUR=β0+β1X1+β2X2+β3X3+……………+βnan

wherePUR = Pedestrian Utilization Rate.Xi-n = explanatory variables.β1-n = are estimated parameters from the model/β0 = constant.

## 6 Results and discussion

The variables involved in this section are rate, guardrails, pelican signals, number of lanes, length and width as summarised in Table 1. The dependent variable involved is rate, which measures the utilization rate (%) of the zebra crossing by pedestrians. It was calculated as the total number of pedestrians who used the zebra crossing divided by the total number of pedestrians crossing the road during a period of time given/recorded. Furthermore, a guardrail is a discrete independent variable which records whether there is any guardrail installed at the road being measured (1: No, 2: Yes). The installation of guardrails near a zebra crossing serves as an obstacle to discourage pedestrians to cross at the mid-point of the road, which is unsafe. Next, the pelican signal is another discrete independent variable, which measures whether pelican signals are installed at the zebra crossing zone (1: No, 2: Yes). The installation of pelican signals can assist the use of zebra crossings among pedestrians by stopping the traffic/vehicle movement for easier and safer road crossing. Next, the number of lanes is a discrete independent variable that measures the number of vehicle lanes at a designated zebra crossing zone. The higher the number of lanes, the higher the number of vehicles going through the roads at a specific period of time. Thus, this leads to a higher traffic flow. Next, the Width of a zebra crossing measured in meters serves as a continuous independent variable. The length of a zebra crossing also indicates the width of a road. Lastly, the width is a continuous independent variable that measures the width of a zebra crossing in meters.

**Table 1:** List of variables (Zebra crossing characteristic).

Variable	Type of variable	Unit or code	Description
Rate	Continuous	%	Utilization rate of zebra crossing by pedestrians
Guardrail	Discrete	1: No	Pedestrian guardrail installation
		2:Yes	
Pelican	Discrete	1: No	Pelican crossing column installation
		2:Yes	
No. of lanes	Continuous	count	The number of vehicle lanes
Length	Continuous	m	Zebra crossing's length or ‘s width
Width	Continuous	m	Crosswalk/zebra crossing's width

**Fig. 2:**
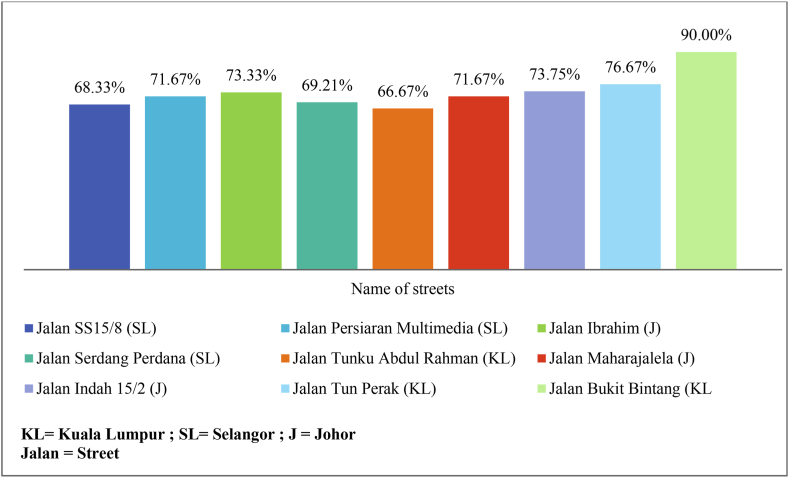
Zebra crossing utilization rate.

Apart from that, the study analysed the zebra crossing utilization rate by calculating the percentage of pedestrians who crossed the road using the zebra crossing as shown in Fig. 2. Studying the figure, it can be noticed that the top three zebra crossing utilization rates were achieved at Jalan Bukit Bintang (KL), followed by Jalan Tun Perak (KL), and Jalan Indah 15/2 (J). On the other hand, the lowest three zebra crossing utilization rates were obtained at Jalan Tunku Abdul Rahman (KL), Jalan Serdang Perdana (SL), and Jalan SS15/8 (SL).

### 6.1 Pedestrians’ utilization rate using MLR models

Multiple regression models were used together with the stepwise method to obtain only the significant variables as shown in Table 2. Based on the table, the F value of 24.721, with a P value of 0.002, indicated that the model is significant at a value of 0.05. Results also showed an R2 value of 0.937, which is close to unity. This suggests that three characteristic/factors in the model were able to explain 93.7% of variance in the zebra crossing utilization rate. Prior to reading the regression weight, it is always a good practice to assess the variance inflation factor (VIF) value. Results showed that all the VIF values were lower than 10, indicating that there is no severe multicollinearity issue for the model.

**Table 2:** Regression weight statistics (Zebra crossing characteristic).

Variable	B	Beta	t	p-value	Tolerance	VIF
(Constant)	19.651		2.712	0.042		
Guardrail	6.85	0.433	2.829	0.037	0.539	1.856
Width	3.398	0.635	5.627	0.002	0.991	1.009
No. of lanes*	3.233	0.409	2.662	0.045	0.535	1.868

DV: Rate, R2 = 0.937, F (3,5) = 24.721, p value = 0.002, *continuous variable.

Out of the five characteristics being tested, only guardrail, Width and the number of lanes were found to significantly affect the zebra crossing utilization rate. Referring to the table, a B value of 6.85 for the guardrail variable indicated that with the installation of guardrails at the zebra crossing zone, the zebra crossing utilization rate will increase by 6.85%. Moreover, the B value of 3.398 for the Width variable showed that each meter increase in the Width of a zebra crossing (width of the road) will increase the zebra crossing utilization rate by 3.4%. In other words, the wider the road, the riskier it is to cross without zebra crossings. Thus, pedestrians would choose to use the zebra crossing in this case. Next, a B value of 3.233 indicated that when the number of lanes increased by 1 lane, the utilization rate of the zebra crossing will increase by 3.2%

Apart from knowing how the characteristics of a zebra crossing affect the utilization rate, the degree of impact was also assessed on the dependent variable. The absolute value of standardised regression weight (BETA) was examined and it was discovered that the Width of a zebra crossing (width of road) has the highest impact on the utilization rate of zebra crossings, followed by guardrail and lastly, the number of lanes.

Based on the results presented in Table 2 above MLR equation can be rewritten as

(2) PedestrianUtilisationRate=19.651+6.85*Guardrail+3.398*Width+3.233*No.oflanes

**Fig. 3:**
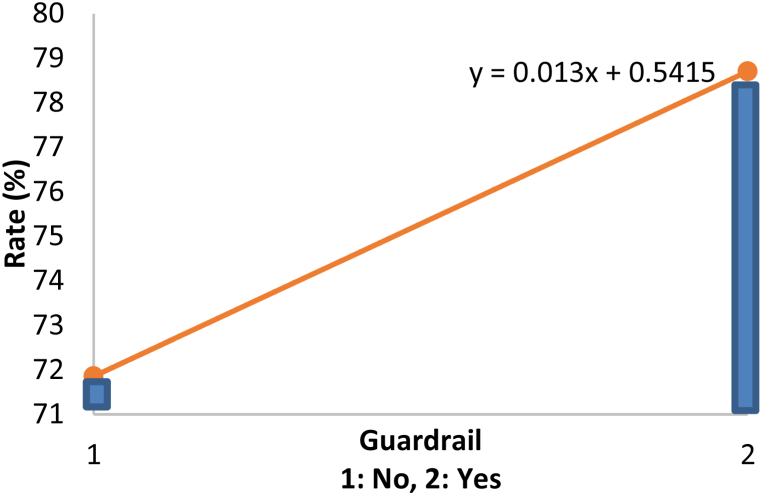
Relationship between guardrail and rate.

**Fig. 4:**
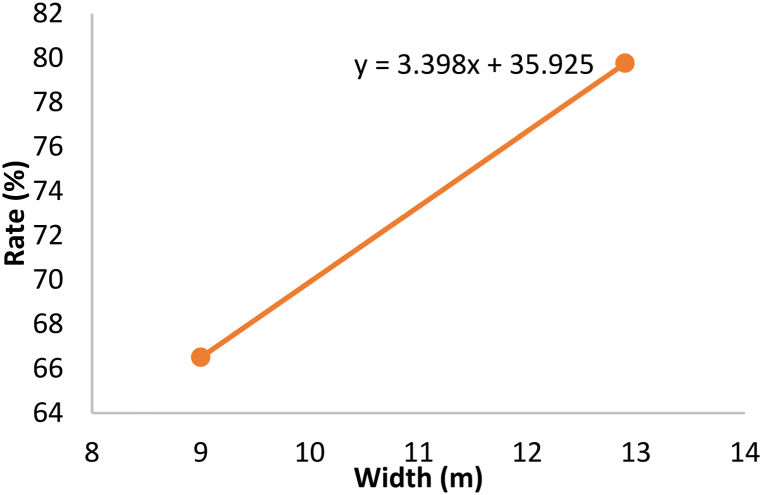
Relationship between Width and rate.

**Fig. 5:**
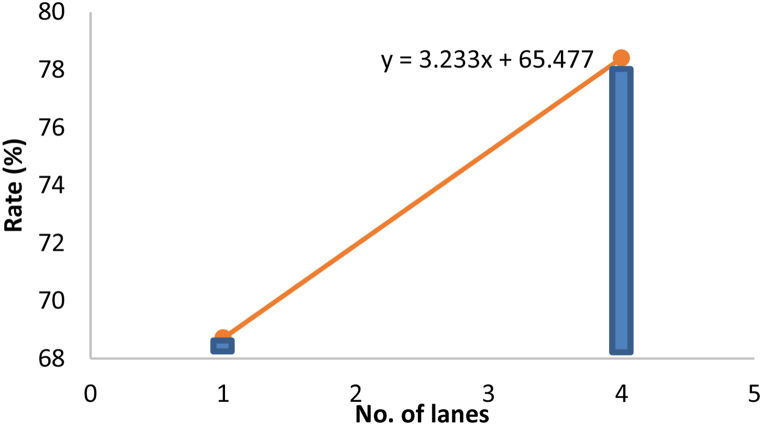
Relationship between number of lanes and rate.

To illustrate the relationship of significant zebra crossing characteristics with zebra crossing utilization rate, Equation (2) was employed to plot the graphs as shown in Fig. 3, Fig. 4, Fig. 5. In order to plot the relationship graph of a particular variable, the other non-related variables will be left constant with their mean values (Guardrail = 1.22, Width = 11.023, number of lanes = 2.44). It was not hard to realise that all of the variables have positive impacts on the rate of zebra crossing utilization.

## 7 Conclusions

This study focused on the utilization level of zebra crossing along urban streets in Malaysia. The usage rate of the facilities by pedestrians at zebra crossing are peculiar with the characteristics of each area and also related to many other factors and understanding of pedestrian towards the available facility. Survey of users and observing pedestrian movement at different 12 locations was used to obtained information needed for this research. The findings revealed that the pedestrian zebra crossing utilization low level are caused by nonexistence of guardrail, number of lanes and Width of zebra crossing. In order to encourage high utilization rate, placement of the zebra crossing and guardrail at appropriate sites, with large roadway width having more than two lanes, have to be considered when providing this facility. The general belief of the result of this study is that it will inform the regulators, pedestrians, drivers and other road users, thereby reducing the risks at vehicular-pedestrian intersections. More so, the findings hope to serve as a benchmark to perpetually increase and adjust road networks in needs of more accurate estimations to reduce the risk and exposure of pedestrians and other road users to accident in several intersections.

## 8 Recommendations

The following recommendations are drawn based upon the results.

1. It is believed from the findings that guardrail installation significantly affects the zebra crossing utilization. Therefore, it is recommended that more guardrails should be installed in areas with high population of pedestrian activities all day long in order to guaranty safety of road users.
2. Zebra crossings should be placed in high densely populated areas such as business and high speed streets. With this, more pedestrians will be encouraged to use it in those areas resulting to high utilization level of the facility and also improving the safety of pedestrians on the road.

## Author contribution statement

Walid Abdullah Al Bargi, Basil David Daniel, Nasradeen A Khalifa, Munzilah Md Rohani, Qinaat Hussain & Rafidah Binti Hamdan: Conceived and designed the experiments; Performed the experiments; Analysed and interpreted the data; Contributed reagents, materials, analysis tools or data; Wrote the paper.

## Data availability statement

Data will be made available on request.

## Declaration of competing interest

The authors have no conflicts of interest to declare. All co-authors have seen and agree with the contents of the manuscript and there is no financial interest to report. We certify that the submission is original work and is not under review at any other publication.

## References

[1] United and Nations (2018). “World Urbanization Prospects The 2018 Revision,”.

[2] Feng S.M., Ding N., Zhang Y. (2010). Proceedings - 2010 WASE International Conference on Information Engineering.

[3] Hamidun R., Ishak S.Z., Endut I.R. (2013). “Assessing pedestrian crossing risk at signalised intersection,”. International Journal of Emerging Technology and Advanced Engineering.

[4] Hoogendoorn S.P., Bovy P.H.L. (2004). “Pedestrian route-choice and activity scheduling theory and models,”. Transp. Res. Part B Methodol..

[5] Harkey D., Zegeer C. (2004). “Pedestrian safety Guide and countermeasure selection system i,”. http://www.walkinginfo.org/pedsafe.

[6] Rizati H., Ishak S.Z., Endut I.R. (2013). BEIAC 2013 - 2013 IEEE Business Engineering and Industrial Applications Colloquium.

[7] Wang X., Tian Z. (2010). “Pedestrian delay at signalized intersections with a two-stage crossing design,”. Transport. Res. Rec.: J. Transport. Res. Board.

[8] Marisamynathan, Perumal V. (2014). “Study on pedestrian crossing behavior at signalized intersections,”. J. Traffic Transport. Eng..

[9] Elvik R. (2000). How much do road accidents cost the national economy?. Accid. Anal. Prev..

[10] Knoblauch R.L., Nitzburg M., Seifert R.F., Richmond Virginia (2001). Stillwater, Minnesota.

[11] Moyano-Diaz E. (2002). Evaluation of traffic violation behaviors and the causal attribution of accidents in Chile. Transport. Res. Part F.

[12] Moyano-Díaz E. (1997). Evaluation of traffic violation behaviors and the causal attribution of accidents in Chile. Environ. Behav..

[13] Fletcher J.P., Sharples J.M. (2001).

[14] Sisiopiku V.P., Akin D. (2003). “Pedestrian behaviors at and perceptions towards various pedestrian facilities: an examination based on observation and survey data,”. Transport. Res. F Traffic Psychol. Behav..

[15] Onelcin P., Alver Y. (2015). “Illegal crossing behavior of pedestrians at signalized intersections: factors affecting the gap acceptance,”. Transport. Res. F Traffic Psychol. Behav..

[16] Al Bargi W.A., Daniel B.D., Muftah M.M. (2017).

[17] Bierlaire M., Antonini G., Weber M. (2003). International Conference on Travel Behavior Research.

[18] Chu X., Guttenplan M., Baltes M. (1878). “Why people cross where they do: the role of street environment,”. Transport. Res. Rec..

[19] Zhang Q., Han B. (2011). “Simulation model of pedestrian interactive behavior,”. Phys. Stat. Mech. Appl..

[20] Li Y., Fernie G. (2010). “Pedestrian behavior and safety on a two-stage crossing with a center refuge island and the effect of winter weather on pedestrian compliance rate,”. Accid. Anal. Prev..

[21] World Health Organization (2013).

[22] Hamidun R., Roslan A., Shabadin A., Ishak S.Z., Voon W.S. (2017).

[23] Lee C., Abdel-aty M. (2005). Comprehensive analysis of vehicle – pedestrian crashes at intersections in Florida.

[24] Demiroz Y.I., Onelcin P., Alver Y. (2015). “Illegal road crossing behavior of pedestrians at overpass locations: factors affecting gap acceptance, crossing times and overpass use,”. Accid. Anal. Prev..

[25] Clifton K.J., V Burnier C., Akar G. (2009). “Severity of injury resulting from pedestrian – vehicle crashes : what can we learn from examining the built environment ?,”. Transport. Res. Part D.

[26] Yang J., Deng W., Wang J., Li Q., Wang Z. (2006). “Modeling pedestrians' road crossing behavior in traffic system micro-simulation in China,”. Transport. Res. Part A Policy Pract.

[27] Naser M.M., Zulkiple A., Al bargi W.A., Khalifa N.A., Daniel B.D. (2017). “Modeling pedestrian gap crossing index under mixed traffic condition,”. J. Saf. Res..

[28] Al Bargi W.A., Daniel B.D., Prasetijo J., Rohani M.M., Nor S.N.M. (2017). MATEC Web of Conferences.

[29] Hamed M.M. (2001). “Analysis of pedestrians' behavior at pedestrian crossings,”. Saf. Sci..

[30] George Y., John G., Eleonora P. (2007). “Modeling crossing behavior and accident risk of pedestrians,”. J. Transport. Eng..

[31] Ulfarsson G.F., Kim S., Booth K.M. (2010). “Analyzing fault in pedestrian–motor vehicle crashes in North Carolina,”. Accid. Anal. Prev..

[32] Zhang G., Yau K.K.W., Zhang X. (2014). “Analyzing fault and severity in pedestrian–motor vehicle accidents in China,”. Accid. Anal. Prev..

[33] Haleem K., Alluri P., Gan A. (2015). “Analyzing pedestrian crash injury severity at signalized and non-signalized locations,”. Accid. Anal. Prev..

[34] Cinnamon J., Schuurman N., Hameed S.M. (Jun. 2011). “Pedestrian injury and human behaviour: observing road-rule violations at high-incident intersections,”. PLoS One.

[35] Rankavat S., Tiwari G. (2016). Pedestrians perceptions for utilization of pedestrian facilities – Delhi, India. Transport. Res. F Traffic Psychol. Behav..

[36] Hasan R., Napiah M. (2017). “The perception of Malaysian pedestrians toward the use of footbridges,”. Traffic Inj. Prev..

